# Next-Generation Intelligent MXene-Based Electrochemical Aptasensors for Point-of-Care Cancer Diagnostics

**DOI:** 10.1007/s40820-022-00845-1

**Published:** 2022-04-11

**Authors:** Arpana Parihar, Ayushi Singhal, Neeraj Kumar, Raju Khan, Mohd. Akram Khan, Avanish K. Srivastava

**Affiliations:** 1 Industrial Waste Utilization, Nano and Biomaterials, CSIR-Advanced Materials and Processes Research Institute (AMPRI), Hoshangabad Road, Bhopal, 462026 MP India; 2 Academy of Scientific and Innovative Research (AcSIR), Ghaziabad, 201002 India

**Keywords:** MXene, Electrochemical devices, POCT, Aptamer, Cancer diagnostics

## Abstract

Delayed diagnosis of cancer using conventional diagnostic modalities needs to be addressed to reduce the mortality rate of cancer. Recently, 2D nanomaterial-enabled advanced biosensors have shown potential towards the early diagnosis of cancer. The high surface area, surface functional groups availability, and excellent electrical conductivity of MXene make it the 2D material of choice for the fabrication of advanced electrochemical biosensors for disease diagnostics. MXene-enabled electrochemical aptasensors have shown great promise for the detection of cancer biomarkers with a femtomolar limit of detection. Additionally, the stability, ease of synthesis, good reproducibility, and high specificity offered by MXene-enabled aptasensors hold promise to be the mainstream diagnostic approach. In this review, the design and fabrication of MXene-based electrochemical aptasensors for the detection of cancer biomarkers have been discussed. Besides, various synthetic processes and useful properties of MXenes which can be tuned and optimized easily and efficiently to fabricate sensitive biosensors have been elucidated. Further, futuristic sensing applications along with challenges will be deliberated herein.

## Highlights

- Shed light on MXene-based electrochemical aptasensors for the detection of cancer biomarkers.
- Strategies for the design and synthesis of biomarker-specific aptamer are presented.
- The properties such as electrical conductivity, chemical stability, mechanical properties, and the hydrophilic–hydrophobic nature of MXenes are discussed.
- Brief insight on futuristic sensing applications along with challenges are highlighted.

## Graphical abstract

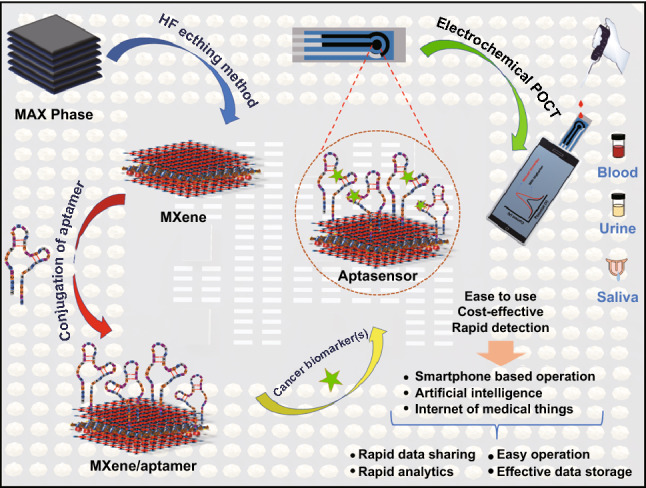

## Introduction

**Fig. 1:**
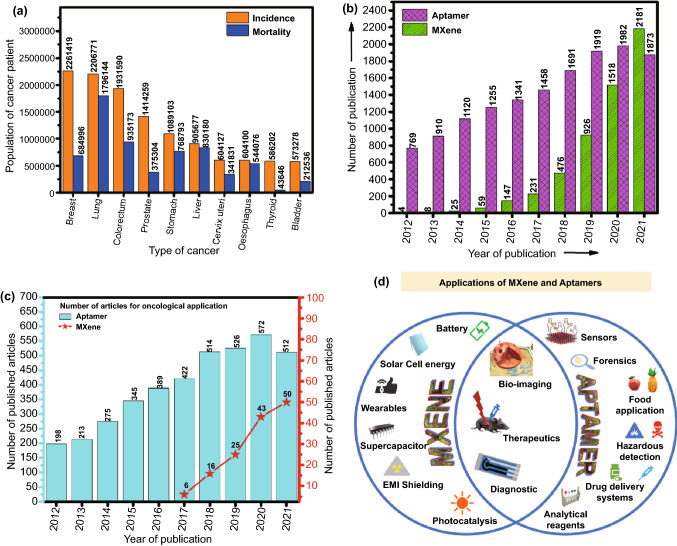
Graphical representation of **a** estimated number of incident cases and mortality worldwide, both sexes and all ages (last access date: 10.12.2021); **b** the literature published on aptamer and MXene in last decade; **c** number of literature published for oncological application of aptamer and MXene in last decade (last access date: 16.12.2021). **d** Applications of MXene and Aptamer

Cancer is one of the major threats to the life and leading cause of death. As per the WHO estimations, cancer is the first or second leading cause of death for people aged below 70 in 112 countries out of the 183 countries and is the third or fourth leading cause in 23 other countries [1]. As per the estimation provided by The International Agency for Research on Cancer (IARC), during the lifetime of a person 1 in 5 people develops cancer. One woman out of 11 women dies with cancer, whereas 1 out of 8 men dies. About 50 million people are living with 5-year history of cancer. Breast, colorectal, lung, cervical, and thyroid cancers are common cancers among women. Lung and prostate cancer are common cancer in men. It was predicted in GLOBOCAN 2020 report that the countries which are identified as low or medium human development index would have the most increase in cancer cases by 2040 [2]. The estimated number of incident cases and mortality associated with various types of cancer is represented in the bar graph in Fig. 1a. The increasing rate of cancer incidence can be controlled if diagnosed at an early stage. Diagnosis at an early stage is quite a difficult challenge because cancer can be asymptomatic which can mislead the diagnosis [3]. Cancer progression is associated with different types of biomarkers. For instance, EGFR, VEGF, HER2, EpCAM, Mucin 1, CEA, CD44 are some important biomarkers found to be associated with the development and progression of cancer [4]. The quantification of these biomarkers can state the stage of cancer progression [5, 6]. By exploring the new technologies and strategies, diseases can be detected at an early stage and can decrease the death rate, and can save lives [7]. Biomarker’s study can be benefited in multiple ways such as risk assessment [8], diagnosis [9], prognosis [10], predicting the treatment efficiency [11], toxicity [12], recurrence of any type of tumor, and many more [13]. The identification of biomarkers associated with a specific type of cancer can help to develop reliable and cost-effective diagnostics to detect cancer at an early stage and monitor it throughout the process of treatment [6, 14, 15]. For cancer diagnosis, conventional techniques such as PET, CT, X-ray, mammography, and tissue biopsy are the mainstream diagnostic modality being still used. However, these techniques failed to detect cancer at an early stage and need a centralized laboratory facility along with trained personnel. Besides, these conventional techniques, molecular techniques such as polymerase chain reaction (PCR), enzyme-linked immunosorbent assay (ELISA), electrophoresis are also used; however, these methods lack accuracy, sensitivity, and selectivity [16].

Recently, biosensors-based advanced diagnostic approaches have shown potential for the early diagnosis of cancer and other deadly diseases [6, 16, 17]. Several techniques employed for the detections are optical [18, 19], electrochemical [20, 21], and piezoelectric [22]. The electrochemical-based detection of several biomarkers such as EpCAM, CD44, VEGF, Mucin 1, CEA has fetched great attention [23]. The electrochemical techniques (amperometry, impede metric, or potentiometric) deliver a highly sensitive rapid and cost-effective platform for early detection of cancer biomarkers. The miniaturization of electrochemical devices helps in their handy usage. Moreover, the electrochemical techniques can reach to attomole detection level and offer high selectivity [24]. Recent studies have shown the crucial role of nanomaterials in the enhancement of the performance of electrochemical devices for the early detection of cancer [23]. The excellent electrical, mechanical, electrochemical, and optical features of 2 D materials have fetched much attention from the past decade in terms of their wide applicability in various fields including disease diagnosis and therapeutics [25]. There are several types of 2D materials such as graphitic carbon nitride [26], transition metal dichalcogenides [27], black phosphorous [28], hexagonal boron nitride (borophene) [29], graphene [30], metal halides [31], metal oxides [32], metal–organic frameworks [33], some polymer [34] which have been investigated for various biosensing applications. Among these, early transition metal carbides and/or nitrides (MXene) are unique in terms of their hydrophilicity, electrochemical, mechanical, and optical properties [35]. They are commonly synthesized using the HF etching method [36]. MXenes are relatively newer when compared to other 2D materials as they were introduced in 2011 at Drexel University. The MXene was usually produced by the etching of the Al element from the MAX phase. MXenes are members of 2D transition metal carbides and carbonitrides [37] and are considered as new generation material currently being used for a wide range of applications [38]. MXene has also been involved in the formation of multifunctional composites such as polymer nanocomposites, carbon nanocomposites, oxide composites.

Recently, MXene has been efficiently used for various biosensing applications [39], targeted drug delivery [40], cancer therapies [41], energy storage [42–44], heat resistance material synthesis [45], catalysis [46], and many other [47]. The MXenes are considered a promising material in analytical chemistry applications owing to their various unique properties [48]. High surface area, high functionalities on the surface, hydrophilicity, production in large batches, high stability, high conductivity, and non-hazardous nature are some of such properties [49]. Moreover, the properties of the MXenes can be tuned accordingly with the changing size, spacing, and thickness of the layers [50]. The MXenes show excellent biocompatibility [51]. MXene-based electrochemical devices display ultra-high sensitivity of detection of target analyte and have shown potential for the detection of cancer biomarkers [52].

**Fig. 2:**
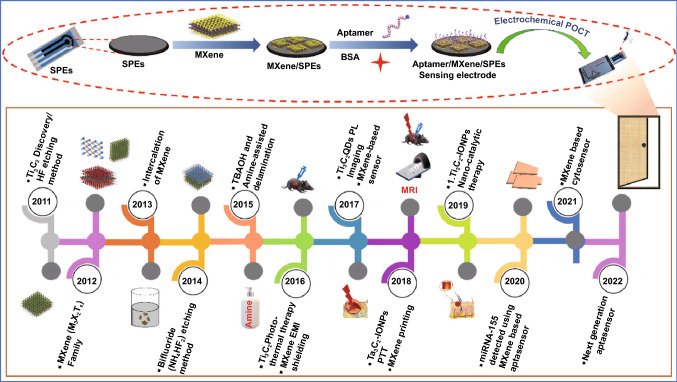
Timeline of MXene for the synthesis and its application in the biomedical field

In the last few years, the use of MXenes has been increased exponentially for various biomedical sensing applications. As per the data collected from Web of Science, the exponential increase was observed in the number of articles that dealt with MXenes and their usage in oncological applications (Fig. 1b, c). However, the biorecognition elements (BREs) play a crucial role in terms of device reusability, repeatability, stability. In this context, conventional biorecognition elements such as an antibody, enzyme, nucleic acid have their limitations in terms of their stability under ambient conditions. The usage of aptamers in various oncological applications has been well reflected by the number of publications as per the data collected from Web of Science (Fig. 1). Among the various BREs, aptamers are preferred. Aptamers are single-stranded DNA, RNA, or peptide sequences that specifically bind to the target molecule [53]. Aptamers offer various advantages over other conventional biorecognition elements as they can be applied to a wide range of targets from small molecules, proteins, viruses to whole cells [54]. Aptamers have increased chemical and thermal stability with high affinity and can also be synthesized easily in large batches. Aptamers are also referred to as chemical antibodies as they function as a chemical substitute to the antibody for diagnostic and detection purposes [55]. Aptamers are carefully chosen from the random pools of sequences using the systematic evolution of ligands by exponential enrichment (SELEX) technique [56]. Aptamers-based biosensors are effectively used in biosensing and other therapeutic application; it is also used for the diagnosis and targeted therapy of cancer [57]. Besides, aptamers are widely used in bioimaging, therapeutics, and diagnosis of cancer [58]. In recent studies, aptamer was used for the detection of cancer biomarkers Mucin 1 [59]; it was also successfully utilized for the blocking/inhibition of SARS-CoV-2 [60]. In another study, aptamer was used to detect the circulating biomarkers in cancer patients’ samples such as proteins, nucleic acids, miRNA in body fluids such as blood, urine, and saliva [61]. Besides, the aptamers platform has been used as a delivery vehicle for targeted drug delivery to the cancerous cells as it possesses various advantages like small size, low immunogenicity, high specificity, and they are flexible that it can easily pass through the solid tumors [62]. Moreover, aptamers have successfully been used for wider applications including environmental monitoring, food analysis, hazardous chemical detection, bioanalytical application, viral detection, biomedical research, and therapeutics. MXenes and aptamer share various common applications including bio-imaging (Fig. 1d) [63], therapeutics [64, 65], and diagnosis [66]. Besides their usage in various other applications, as shown in Fig. 1d. The timeline showing the evolution of usage of MXene since its discovery in 2011 to the fabrication of MXene-based aptasensor for cancer diagnostics and therapeutics is shown in Fig. 2. Additionally, the schematic for the fabrication of IoT-enabled smartphone-based electrochemical aptasensors using MXene is shown in Fig. 2. The use of biomarkers along with the aptamer using electrochemical detection technique can be exploited as a boon towards early cancer diagnostics [66–69].

In the present review, we gathered information regarding MXene-based electrochemical aptasensors for the detection of cancer biomarkers. The potential cancer biomarkers for which aptamers are available along with approaches for designing and synthesis biomarker-specific aptamers have been discussed. Various synthetic techniques, as well as post-processing modification of MXenes, have been elucidated, which can be helpful for the development of selective and sensitive aptasensors. In addition, emphasis has been given to properties such as electrical, optical, thermal, chemical stability, and mechanical properties of MXenes. Furthermore, a brief insight over futuristic sensing applications of MXene, as well as difficulties and perspectives, has been presented. The content of this review is expected to improve knowledge and pave the way for the development of future next-generation electrochemical biosensors which could revolutionize the field of disease diagnosis.

## Potential Biomarkers Associated with Cancer

The cancer cells grow rapidly which often led to tumor cell heterogeneity because it exhibits different morphology and behavior. Tumor heterogenicity can be identified using reliable biomarkers. A biomarker is an abbreviation for biological markers associated with a specific disease, and it is measured as an indicator of the extent of clinical diseases [20, 70]. The presence of a biomarker in blood or any other body fluid or tissues indicates the particular condition of disease [71]. Biomarkers can be classified into subtypes based on their application as diagnostic biomarkers, monitoring biomarkers, pharmacodynamic/response biomarkers, predictive biomarkers, and prognostic biomarkers [72]. Diagnostic biomarker assists in knowing disease development and the accomplishment of the treatment. The diagnostic biomarker not only recognizes the person with a certain disease but also classifies the disease [73]. Monitoring biomarkers are used multiple times for evaluating the disease status or patient’s condition in response to pharmaceuticals or any other external agent [74]. The pharmacodynamics/response biomarker is those biomarkers whose level changes in response to medicine or any other environmental factors [75]. The predictive biomarkers are used to recognize people which are more likely to be affected either positively or negatively by specific medical products or any other external agents [76]. A prognostic biomarker is a biomarker that predicts the probability of the occurrence of a clinical condition, its recurrence, or progression in a certain population [33]. Cancer biomarkers can be employed for early diagnosis of a tumor or its reappearance, for prognosis or predicting a patient’s response to specific drugs or treatment, or knowing the toxicity of therapeutic interventions [77]. The valuation of biomarkers can be influenced by some factors such as type of tumor whether it is new or recurrent, tumor heterogeneity, and treatment effect [78]. The malignancy and metastasis pathways are the major barriers that limit the conventional therapeutics strategies. Due to tumor heterogeneity, the expression of cancer biomarkers can differ between biopsy tissue and surgical resection specimens in a patient with untreated newly diagnosed cancer. Further, cancer treatment can also cause a change in the expression of biomarkers and the emergence of resistant cancer cells that survive and become prevalent following each treatment. Because tumor DNA is fundamentally unstable, it can change over time, resulting in differences between initial and recurrent/persistent tumors. Because of the primary tumor’s treatment and the innate instability of tumor DNA, the molecular phenotype and the biomarkers of primary vs. recurring malignancies can differ [78].

Biomarkers associated with various diseases including cancer can be detected through various approaches. The general approach depends on the basic biology of the tumor and surroundings [79]. With the advancements in technology and more knowledge about the tumor, biomarkers can be identified easily and rapidly using different techniques [80]. Some of the techniques used for the identification and quantification of the biomarker are advanced sequencing, gene expression arrays, and mass spectroscopy [81]. The major challenge is that these techniques produce a vast amount of data that needs to be analyzed. The more focus is on the development of such techniques which can deliver accurate results and avoid further validation.

Various other analytical techniques such as polymerase chain reaction (PCR), immunohistochemistry, flow cytometry have been used for the evaluation of cancer biomarkers; however, they lack acceptable sensitivity and need sophisticated instrumentation facilities with long run time. Biosensors-based detection of biomarkers can be used efficiently for the early diagnosis of cancer disease [15, 20, 23]. The use of biorecognition elements (BREs) such as an antibody, aptamers, enzymes with conjugation to biomarkers associated with cancer cells enhanced the selectivity and sensitivity of the detection technique [82]. The use of aptamers over conventional BREs biorecognition elements gives promising results with increased selectivity and sensitivity. Moreover, aptamers over other BREs such as antibodies, enzymes, cells are physically, chemically stable, and also, regenerated without losing integrity. Aptamers can be synthesized for a wide range of analytes with high specificity [83]. To date, a variety of aptamers with the ability to bind to the receptors of the once cells have been employed. These include prostate-specific membrane antigen (PSMA), Mucin 1 (MUC1), protein tyrosine kinase-7 (PTK7), VEGF, CA-125, CEA, CD44, and IL-6, etc. [84]. Aptamers designed for the identification and detection of various biomarkers associated with different types of cancer are enlisted in Table 1 along with their sequences and their properties.

**Table 1:** Cancer biomarkers associated with different types of cancer for which aptamers have been designed and exploited

S. no.	Cancer biomarker	Cancer type	Function and properties	Aptamer sequence	References
1	VEGF- Protein	Breast cancer	Promotes angiogenesis Prognosis	5′-GGG CCG TTC GAA CAC GAG CAT GGT GGG TGG TGG CCC TAG GAT GAC CTG AGT ACT GTC C-3′	[85]
				5′-GCA GCT ATG TGG GGG ACG TCC AGC TGC-FAM-3′ 5′-TGG ATA CGG CCG GGT AGA TA-3′	[86]
2	PCA3- Protein	Prostate cancer	Prognosis	5′-AGUUUUUGCGUGUGCCCUUUUUGUCCCC-3′	[87]
	PSA	Prostate cancer	Cleave semenogelins in the seminal coagulum	5′-SH-(CH2)6-TTTTTTTTTTATTAAAGCTCGCCATCAAATAGCTGC-3′ 5′-SH-(CH2)6-TTTTTTTTTTGCAGCTATTT-Cy5-3′ AATTAAAGCTCGCCATCAAATAGCTTT GAGCGGGGTTGCTGGGATGATAAGGCCCCTTTGATGTCTG	[88]
				5′-GAGCGGGGTTGCTGGGATGATAAGGCCCCTTTGATGTCTG-3′	[89]
				5′ -NH2-(CH2)6-TTT TTA ATT AAA GCT CGC CAT CAA ATA GCT TT-3′	[89] [90]
				5′-CCGUCAGGUCACGGCAGCGAAGCUCUAGGCGCGGCCAGUUGC-3	[91]
				5′-HS-(CH2)6-ATT AAA GCT CGC CAT CAA ATA GC-3′	[92]
3	MUC1	Colon, breast, ovarian, lung, and pancreatic cancer	Protect cells from infection	5′-NH2-GCAGTTGATCCTTTGGATACCCTGG- 3′	[93]
4	CEA	Colorectal cancer, esophageal cancer, gastric carcinoma, pancreatic carcinoma	Cancer diagnosis and treatment	5′-Texas Red -ATACCAGCT TATTCAATT-3′, random ssDNA 5′-TCATTACATGTTTCCT TACTTC CAG-3′	[94]
				SH-ATACCAGCTTATTCAATT	[95]
				5′-ATACCAGCTTATTCAATT-3′	[96]
5	EpCAM	Colorectal, breast, gallbladder, pancreatic, liver cancer	Cancer diagnosis, prognosis, and therapy	5′-/5carboxy1/-CAC TAC AGA GGT TGC GTC TGT CCC ACG TTG TCA TGG GGG GTT GGC CTG-3′	[97]
6	EGFR	Gastric, breast, ovarian, and colorectal cancers	Promotes cell division and proliferation	5′-TAC CAG TGC GAT GCT CAG TGC CGT TTC TTC TCT TTC GCT TTT TTT GCT TTT GAG CAT GCT GAC GCA TTC GGT TGA C-3′	[98]
7	IL-6	Lung cancer	Cytokine biomarker regulates immune responses	5′-NH2- GGT GGC AGG AGG ACT ATT TAT TTG CTT TTC T -3′	[99]
		Lung and breast cancer	Radiation injury biomarkers	5′ -SHC6- TTTTT GGGGG AAAAA CTTCCAACGCTCGTATTGTCAGTCTTTAGT-3′	[100]
8	HER2	Breast, gastric cancer	Downregulation of HER2 can induce apoptosis by altering cell proliferation and downstream signaling pathways	5′-GCAGCGGTGTGGGG-3′	[101]
				5′-NH2-(CH2)6-GGG CCG TCG AAC ACG AGC ATG GTG CGT GGA CCT AGG ATG ACC TGA GTA CTG TCC-3′	[102]
				5′-biotin-ACGACCCGATAAGTGCATTAGCACGTCCGAGAAAGGCCAGACGGGTCACACAGAGTTA-3′	[103]
				5′-SH-(CH2)6-ATTAAGAACCATCA CTCTTCCAAATGGATATACGACTGGG-3′	[104]
				5′ -TCT AAA AGG ATT CTT CCC AAG GGG ATC CAA TTC AAA CAG 6 S–S-3′	[105]
				[ThiC6]AACCGCCCAAATCCCTAAGAGTCTGCACTTGTCATTTTGTATATGTATTTGGTTTTTGGCTCTCACAGACACACTACACACGCA	[106]
9	HE4	Ovarian cancer	Early detection	5′-FAM-AGC AGC ACA GAG GTC AGA TG-3′, reverse primer 5′-biotin-TTC ACG GTA GCA CGC ATA GG-3′, 5′-FAM-AGC AGC ACA GAG GTC AGA TG (N)25 CCT ATG CGT GCT ACC GTG AA-3′	[107]
10	CA125	Ovarian cancer	Early diagnosis	5′- AAAAAACTCACTATAGGGAGACAAGAATAAACGCTC AA-3′	[108]
11	CRP	Cancer	Diagnose inflammatory reactions in cancer	5′ -CGAAGGGGATTCGAGGGGTGATTGCGTGCTCCATTTGGTGTTTTTTTTTTTT-(CH2)6-NH2-3′ 5′-CGAAGGGGATTCGAGGGGTGATTGCGTGCTCCATTTGGTGTTTTTTTTTTTT-(CH2)6-SH-3′	[109]
12	AFP	Hepatocellular, prostate, and ovarian cancer		5′-GGCAGGAAGACAAACAAGCTTGGCGGCGGGAAGGTGTTTAAATTCCCG GGTCTGCGTGGTCTGTGGTGCTGT-3′	[96]
				5′-HS-(CH2)6- GTG-ACG-CTC-CTA-ACG-CTG-ACT-CAG-GTG-CAGTTC-TCG-ACT-CGG-TCT-TGA-TGT-GGG-TCC-TGTCCG-TCC-GAA-CCA-ATC-3′	[110]
13	CTCs	Hepatocellular cancer,	Used to evaluate cancer metastasis	5′-dithiol-TTTTTTTTTTACAGCATCCCCATGTGAACAATCGCATTGTGATTGTTACGGTTTCCGCCTCATGGACGTGCTG-3′ ZY5C aptamer 5′-SHC6TTTTTTTTTTCACGCATAGCCTTTGCTCCTCGTCTGGAACGTCGCAGCTTTAGTTCTGGGCCTATGCGTG-3′	[111]
		Ovarian cancer		5′ -GCAGGAAGACAAACA-N40-GGTCTGTGGTGCTGT3′	[112]
				5′-SHCACTACAGAGGTTGCGTCTGTCCCACGTTGTC ATGGGGGGTTGGCCTG	[113]
14	Tg	Thyroid cancer	Diagnosis and postoperative monitoring	Primer sequences (forward 5′ -CCTAACCGATATCACACTCAC-3′, reverse 5′ -GATACTCCAATGACGACCAAC-3′) and the random ssDNA library (82 nt, 5′ -FAM-CCTAACCGATATCACACTCAC-N40- GTTGGTCGTCATTGGAGTATC-3′)	[114]
15	NCL	Prostate cancer	Regulation of several mechanisms related to nucleic acid metabolism and tumor prognosis	5′—FAMGTTGGGGTGGTGGTGGTTGTGGTGGTGGTGGCCAAC-Dabcyl -3′	[115]
				5′-GGTGGTGGTGGTTGTGGTGGTGGTGG-Rox-3ˊ	[116]
16	AGR2	Pancreatic, breast, ovarian, prostate, and colorectal cancer	Early diagnosis	5′-CG_3_TG_3_AGT_2_GTG_9_TG_3_AG_3_T_2_-3′	[117]
17	STIP1	Ovarian cancer	Prognosis	5′-ATCCAGAGTGACGCAGCA CGGCACTCACTCTTTGTTAAGTGGTCTGCTTCTTAA CCTTCATCGACACGGTGGCTTA-3′	[118]
18	lncPCA3	Prostate cancer	Predict prostatic biopsies	5′- AGUUUUUGCGUGUGCCCUUUUUGUCCCC-3′SH	[119]
19	PDGF-BB	Breast, pancreatic, prostate, ovarian, and liver	Play a potent role in the growth and metastasis	5′-C_6_-CAG GCT ACG GCA CGT AGA GCA TCA CCA TGA TCC TG-3′	[120]
20	KIT	Cancer		5′-GAG GCA TAC CAG CTT ATT CAA GGG GCC GGG GCA AGG GGG GGG TAC CGT GGT AGG ACA TAG TAA GTG CAA TCT GCG AA-3′	[121]
21	PTK7	Breast, lung, colon, and gastric cancer	plays important role in cell regulation, ion transport, and cancer development	5′-H2N-ATC TTA CTG CTG CGC CGC CGG GAA AAT ACT GTA CGG TTA GAT TTT TTT TTT-3′	[122]
22	Thy-1	Pancreatic ductal adenocarcinoma	Facilitate the attachment of tumor cells to endothelial cells and promote tumor metastasis	5′-CAGGGGACGCACCAAGG-TTGCCCACAGAWCYGTGGAAGCCGAACCGCGTGCWAGXCGYG-CCATGACCCGCGTGCTG-3′ 5′-CAGGGGACGCACCAAGG-TTGCCCACCYCCCYGTGCGGGCCACAGAGCAGCAGTGXCGYG- CCATGACCCGCGTGCTG-3′ 5′-CAGGGGACGCACCAAGG-TTGCCCACCGWACYGTGCAGGXCGAACTACAGGCACGXCGYG- CCATGACCCGCGTGCTG-3′	[123]

## Approaches for Designing of Aptamers

**Fig. 3:**
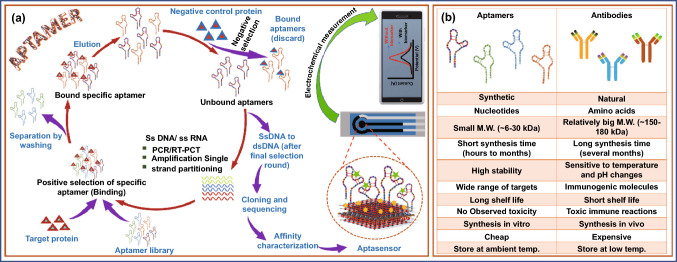
**a** Synthesis method for aptamer and detection through electrochemical method, **b** comparison of aptamers and antibodies as biorecognition element

Aptamers are single-stranded synthetic nucleic acids (DNA or RNA sequences) that selectively binds with specific biomarker and can be wrapped into 2D (two-dimensional) and 3D (three-dimensional) structures. Due to 2D and 3D structures, they possess enhanced surface density and reduced spatial blockage and thus show high binding performance toward the target [82, 122–124]. Aptamers are robust molecules in terms of structure and functional aspects and hence remain stable throughout a wide variety of temperatures and other stringent conditions. Unlike antibodies, which need the biological systems for their synthesis, aptamers can be synthesized chemically, remain stable in the pH range of 2–12, and can undergo heat refolding. Another advantage of aptamers is that they can be chemically modified by adding functional groups to match the target molecule’s detection requirements [125]. Aptamers can be generated from oligonucleotide libraries via an in vitro selection technique called SELEX (Systematic Evolution of Ligands by EXponential Enrichment) [125, 126]. In this process, the aptamers have been selected via an iterative process that involves binding of the target protein/biomarkers with an oligonucleotide in a library followed by washing unbound aptamers and amplification of bound selected oligonucleotide. Multiple cycles of the SELEX process led to the enrichment of highly specific oligonucleotides against a particular target. Afterward, the aptamer with high selectivity was selected, affinity-purified, and sequenced to produce a specific aptamer library [127]. The steps of aptamer synthesis via the SELEX process are shown in Fig. 3a. Cell-SELEX, microfluidics-SELEX, capillary electrophoresis-based SELEX, FACS-based SELEX, magnetic bead SELEX, microtiter plate-SELEX, and in vivo SELEX are a few of the SELEX variations that have recently been developed for the improved synthesis of aptamers [127–129]. Figure 3a, b depicts the fabrication of aptasensors along with the advantages of using aptamers over antibodies. The most common biosensor-based diagnostic approaches are optical, electrochemical, and piezoelectric. These biosensors are classified as labeled or label-free aptasensors based on the transduction processes used. The electrochemical sensing techniques can be used for both label-free optical sensors as well as label-based aptasensors [130]. The details of electrochemical aptasensors their design and fabrication strategies are discussed in Sect. 5 of this review.

## MXene: Synthesis and Properties

Owing to the wide application of MXenes', its intrinsic compositional properties are necessary to evolve in distinct directions. These properties, on the other hand, are determined during the synthesis stage and are influenced by a variety of parameters, including the precursor MAX phase, the reaction duration, the etchant, and the temperature of the process. The regulation of these parameters remains a challenge to get appropriately designed MXenes with desired characteristics [131]. Conventional MXene, o-MXene, and i-MXene are the types of MXene [132]. MXenes are produced by a process of selective etching in appropriate solvents or solutions. Etching is generally carried out in acidic solutions [133]. Etching results in surface terminations with the various functional groups making them feasible to be used further [133, 134]. Due to the strong mechanical, optical, and electrical properties, MXene's have attracted attention for a wide range of applications in energy, medicines, and diagnostics [135].

### Structure and Synthesis of MXene

**Fig. 4:**
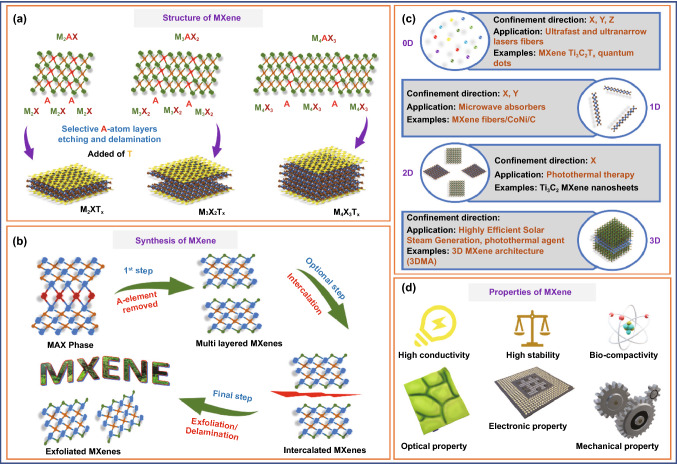
**a** Structure of MXene. **b** Synthesis of MXene. **c** Dimensional-based classification. **d** Properties of MXene

After the discovery of MXene a decade ago, it has gained considerable attention in the research field [37]. The general formula for MXene is M_n+1_X_n,_ while for MAX phase the general formula is M_n+1_AX_n,_ where *n* = 1, 2, or 3, M is an early transition metal (Ti, V, Nb, Mo, Cr, Ta, Hf), A is group 13 or 14 elements, and X is mostly C and/or N [136]. There are about 70 MAX phases known (such as Ti_2_AlC and Ti_3_AlC_2_), and many new combinations are being discovered day by day (e.g., the quaternary ordered MAX phases) and related materials [130, 131, 136]. In the MAX phases, M_n+1_X_n_ are the stable layers, whereas the A layer is comprised of weaker bonds. The etching of the Al layer from the MAX phase (Ti_3_AlC_2_) gives rise to Ti_3_C_2_ (MXene) [137]. The suffix “ene” to the MXene shows that MXene properties are similar to another popular 2D material graphene [138]. MXenes possess excellent physical and chemical properties including low density, high hardness, good resistivity against corrosion, high conductivity [139]. The various compounds of MXenes can be categorized into different categories based on the complexity of the structure. The MAX phase is an MXenes precursor with the stoichiometry M_n+1_AX_n_, where *n* = 1, 2, or 3, and “M” is a metal which belongs to the d-block transition metal family, “A” is a group 13 or 14 elements (e.g., Si, Ge, Al, or Sn), and “X” can be carbon, nitrogen, or both. The layers “M” and “A” are intercalated in between phases, which have a hexagonal structure. The “X” atoms occupy the octahedral positions created by the “M” elements [44]. A detailed insight into the structure of MXene is shown in Fig. 4a. The removal of "A" elements from the MAX phase led to the production of multi-layer MXenes which upon intercalation produces intercalated MXene that can be exfoliated or undergo delamination. The steps are depicted in Fig. 4b. Taking the properties of MXene into consideration, recent studies have employed all OD, 1D, 2D, and 3D dimensions of MXene. For instance, 0D MXene Ti_3_C_2_T_x_ quantum dots used for ultra-fast and ultra-narrow laser fibers manufacturing [140], 1D MXene fibers/CoNi/C has been used as microwave absorbers [141], 2D Ti_3_C_2_ MXene nanosheets for biosensing and photothermal therapy [137], and 3D MXene architecture (3DMA) used for highly efficient solar steam generation (Fig. 4c) [142]. The accessibility of a large surface area of material for interaction, selective binding, and the ability to transduce the binding of analyte into the recognizable signal is the ideal properties of sensing material. The 2D materials exhibit a large surface-to-volume ratio when compared to 0D, 1D, and 3D analogs for material analyte interaction which ensures high sensitivity at an extremely low concentration of target analyte [143]. Their unique features, which result from a rare blend of ceramic and metallic behaviors, have grabbed much research interest. MAX phases have high hardness, low density, and high corrosion resistance, akin to ceramics, while also having high electrical and thermal conductivities and enhanced machinability which mimics metallic material [143, 144]. A schematic of desirable properties of MXene is depicted in Fig. 4d.

The fundamental bonds are responsible for these revolutionary properties, whereas M–X bonds include a mixture of ionic and covalent interactions. The nature MA bonds are entirely metallic. As a result, unlike other 3D layered materials like transition metal dichalcogenides and graphene, which have weak interactions, MAX phases have robust bonds that provide stability and inhibit cleavage by shearing or other mechanical means. Initially, the chemical exfoliation method allowed the creation of MXene-based 2D materials from primary bonded MAX phases [37].

**Fig. 5:**
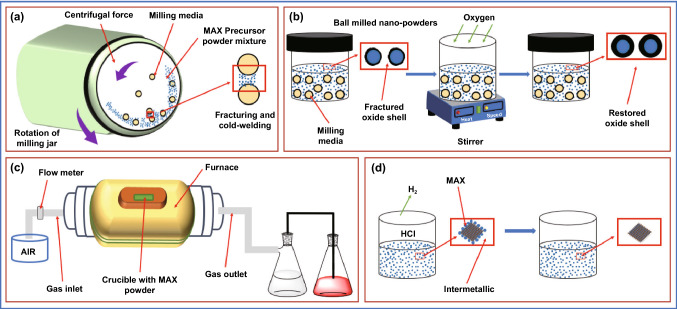
Steps associated with the production of the MAX phase: **a** ball milling, **b** passivation by oxygen, **c** high-temperature synthesis, and **d** acid washing to remove interferents

Due to low cost, simplicity, and scalability, high-temperature synthesis of MAX phase from binary elements is the most commonly used procedure. In this method, TiC, Ti, and Al powders are mixed in ball milling (Fig. 5a, b) and then annealed in a tube furnace under an inert atmosphere in presence of argon at 1400 °C for 2 h with a rate of heating and cooling of 3 °C min^−1^ (Fig. 5c). Afterward, the material was ground, milled, and drilled using pestle mortar followed by sieving to yield a powder of known particle size. The powder was subjected to HCL wash to remove impurities (metallic and intermetallic) before sieving (Fig. 5d) [145].

**Fig. 6:**
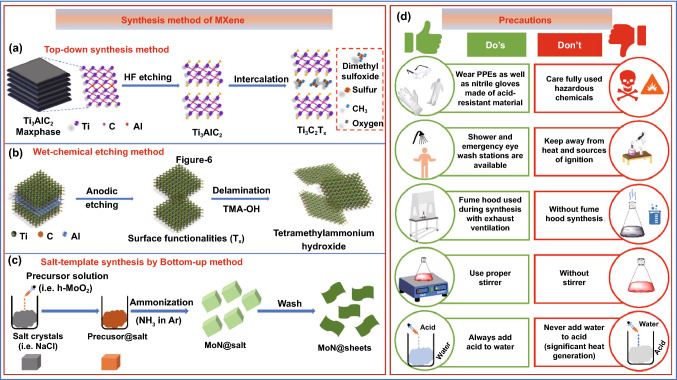
Synthesis method of MXene. **a** Top-down synthesis method, **b** wet chemical etching method, **c** salt-template synthesis by the bottom-up method, **d** safety measures and general instructions for laboratory

Various synthesis techniques have been introduced which contributed significantly to the field of MXenes’ research to meet the appropriate requirement for various applications. Etching and delamination are extensively used as prime methods for the synthesis of MXenes [146]. In the precursor MAX phase, the etching procedure is primarily used to disrupt the M–A metal bond. Fluorine-containing acid etching [34, 147], halogen etching [148], strong alkaline etching [149], high-temperature etching [150], and electrochemical etching [151] are the different types of etching methods used so far. The most popular technique for etching the A layer is to use a hydrofluoric acid (HF) or a strong alkali. Many sagging bonds arise on the surface of 2D MXene nanosheets as a result of this process, which is converted into numerous terminations in groups such as –F, –O, and –OH. In the meantime, the MXene nanosheets are exposed to more or lesser flaws during the reaction, making it easier for the material to deteriorate and lose its original properties [152], while in the delamination method, under the influence of mechanical force [37] or chemical intercalants [153], multilayer MXenes peel apart to generate single- or few-layer lamellae in the delamination stage. The extreme vibrations caused by ultrasound, on the other hand, cause a shrink of the size of the MXene nanosheet which led to a loss of electrical and mechanical characteristics. Chemical intercalation allows MXenes to be reduced in size to some extent, but it is tough to eliminate these chemical agents in successive operations, which affect the conductivity of MXenes films significantly [154]. As a result, MXenes’ real properties deviate significantly from their theoretical values, restricting their uses in several areas such as energy storage [155], catalysis [156–158], electromagnetic shielding [40, 45, 48, 158, 159], flexible electronics [160], and chemical sensing [161]. Based on literature several methods of MXene synthesis can be categorized into top-down [133], wet chemical [148], and bottom-up [162] approaches. The detailed insight of each method is illustrated in Fig. 6. The top-down method is the most preferred approach for the synthesis of MXene. In this method, etching of A elements from the 3D, MAX phase was carried out using HF (Fig. 6a), while in the bottom-up method the MXene is constructed using small organic or inorganic molecules (Fig. 6b). The bottom-up method provides the advantage of controlled synthesis with appropriate size and morphology and surface terminations when compared to the top-down method. Further, in the wet chemical method, anodic etching was performed followed by delamination (Fig. 6c). The precautions taken during the synthesis of the MAX phase and MXene remain a concern among the scientific community as it uses acid and high temperature. The safety measures such as PPEs kit, gloves, fume hood, and proper handling of acid and water while synthesis of MXene should be properly taken care of. The safety measures should be followed in general, while acid and other corrosive chemicals are shown in Fig. 6d.

**Fig. 7:**
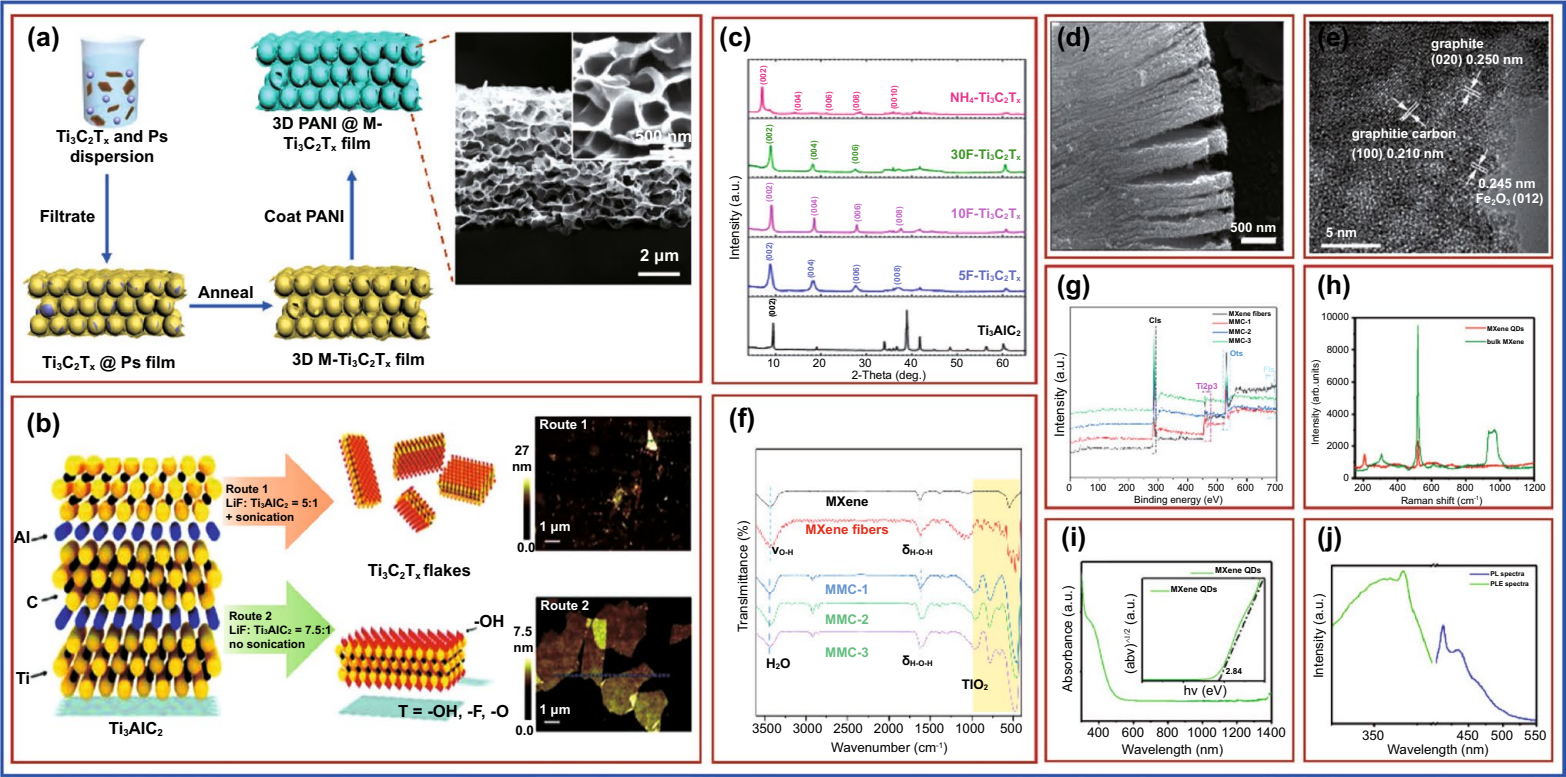
**a** Schematic representation of the preparation of 3D macroporous PANI@M-Ti_3_C_2_T_*x*_ frameworks with PS spheres as a template, inset showing SEM images of the formed 3D PANI@M–Ti_3_C_2_T_*x*_ film. Copyright from Ref. [163]. **b** Synthesis of Ti_3_C_2_T_x_ via two different routes with or without sonication, inset showing AFM images of the synthesized Ti_3_C_2_T_x_ flakes. Copyright from Ref. [164]. **c** XRD patterns of Ti_3_AlC_2_ powder and Ti_3_C_2_T_x_ MXene powders synthesized with 5, 10, and 30%wt HF and in situ HF by using NH_4_-Ti_3_C_2_T_x_ routes. Copyright from Ref. [165]. **d** The SEM image of MXenes at 500 nm. Copyright from Ref. [166]. **e** HR-TEM images of Ti_3_C_2_T_x_@FePcQDs hybrid structure. Copyright from Ref. [167]. **f** FTIR spectrum, **g** XPS spectra of various samples. Copyright from Ref. [141]. **h** Raman spectra of bulk MXene and MXene quantum dots. **i** UV–Vis absorption spectrum of MXene QDs; the inset showing the value of the bandgap fitted. **j** Excitation and emission spectra of MXene QDs. Copyright from Ref. [140]

An ultrafast polyaniline@MXene cathode was created by casting a homogeneous polyaniline layer onto a 3D porous Ti_3_C_2_T_x_ MXene; by Li et al., PS spheres with a negative surface charge can disperse homogeneously in water with the same negatively charged Ti_3_C_2_T_x_ MXene flakes (Fig. 7a), which can then be vacuum-assisted filtered into a flexible PS@Ti_3_C_2_T_x_ film (6 m) with Ti_3_C_2_T_x_ MXene flakes wrapping the PS spheres’ surface (500 nm). A freestanding and flexible 3D microporous Ti_3_C_2_T_x_ (3D M-Ti_3_C_2_T_x_) film with an open and interconnected structure was developed after eliminating the PS by thermal annealing at 450 °C in argon. This film displayed an electrical conductivity of 600 S cm^−1^, which is higher than a 3D graphene film with a comparable structure (12 S cm^−1^) (Fig. 7a) [163]. Lipatov et al. described a new synthetic approach for making high-quality monolayer Ti_3_C_2_T_x_ flakes. In this work, two varieties of Ti_3_C_2_T_x_ flakes were created. Ti_3_C_2_T_x_ was manufactured following the Route 1 method, which involved soaking Ti_3_AlC_2_ powder in a LiF-HCl solution with a molar ratio of LiF to MAX of 5:1. This approach produces mostly monolayer flakes. The molar ratio of LiF to MAX was increased to 7.5:1 in the modified technique in the method opted in Route 2 which provides an excess of Li^+^ ions for intercalation. Herein, the HCl to LiF ratio was doubled to enhance aluminum etching. Further, the delamination of Ti_3_C_2_T_x_ particles created using Route 2 did not require sonication. The key distinctions between Routes 1 and 2 along with the atomic force microscopy (AFM) images which were used to examine the thickness and morphologies of the flakes produced by both procedures are shown in Fig. 7b. The AFM images revealed that the Ti_3_C_2_T_x_ flakes synthesized by Route 2 are much larger than those produced by Route 1 (Fig. 7b) [164]. Alhabeb et al. produce titanium carbide (Ti_3_C_2_T_x_), the most researched MXene, utilizing several etchants and delamination processes. They also discuss the implications of synthesis settings on Ti_3_C_2_T_x_ size and quality, as well as the best procedures for the application. Low concentrations of HF (5 wt%) for 24 h were shown to be just as effective as higher concentrations (10 wt. percent HF for 18 h and 30 wt. percent HF for 18 h), as confirmed by energy-dispersive X-ray (EDX) analysis and X-ray diffraction (XRD) patterns (Fig. 7c) by a shift of the (002) peak of Ti_3_AlC_2_ from 9.5° to 9.0° for Ti_3_C_2_T_x_ and no residual Ti_3_AlC_2_ peaks after etching for 5, 10, and 30F–Ti_3_C_2_T_x_. Further, the elimination of Al in Ti_3_AlC_2_ and the insertion of surface terminations (expressed as Tx) in Ti_3_C_2_T_x_ (e.g., –F, –O, –OH) result in a reduced peak shift of the basal planes ((002) peak) [165]. The MXenes were characterized using scanning electron microscopy (SEM). As shown in Fig. 7d, the MXenes had a dense layer and an accordion-like shape [166]. The high-resolution transmission electron microscopy (HR-TEM) picture of the Ti_3_C_2_T_x_@FePcQD nanohybrid (Fig. 7e) revealed interplanar distances of 0.25, 0.245, and 0.21 nm, respectively, corresponding to the (020) plane of graphite, the (012) planes of hexagonal Fe_2_O_3_, and the crystallographic (100) plane of graphitic carbon. In the synthesis of FePc QDs, Fe_2_O_3_ nanoparticles and carbon dots were produced concurrently at 180 °C. The findings suggested that FePc QDs and Ti_3_C_2_T_x_ nanosheets can be successfully integrated [167]. FTIR spectra of MXene and MXene fiber are shown in Fig. 7f. The X-ray photoelectron spectroscopy (XPS) curves of each group of samples are shown in Fig. 7g. The typical peaks of C 1* s* (285 eV), Ti 2*p* (459 eV), and O 1* s* (530 eV) were visible [141]. The bulk gene and MXene QDs were also characterized using Raman spectroscopy. From 200 to 1100 cm, the spectrum has six conspicuous peaks (Fig. 7h), which is consistent with earlier findings. The peak of about 500 cm, on the other hand, represents the signal of the Si substrate, as shown in several test results. The optical quality of MXene Ti_3_C_2_T_x_ QDs as investigated using UV–Vis absorption spectroscopy is shown in Fig. 7i. The bandgap is calculated as 2.84 eV by mapping the absorption to the band edge. A fluorescence spectrometer for determining the PLE spectra of MXene QDs and testing their fluorescence properties is displayed in Fig. 7j. As stimulated at 367 nm, the brightest peak appears about 415 and 430 nm [140].

### Properties of MXene

High Young’s modulus, a tunable bandgap, thermal and electric conductivities are some of the distinctive MXene features. The hydrophilic surfaces of MXenes along with high electrical and thermal conductivities set them apart from the majority of 2D materials [148]. Eventually, specific composition and involvement of different transition metals “M” and “X” elements, and varied functionalization of the surface via chemical and thermal processes led to structure/morphological changes, which can be used to tune their properties and applications performances [163]. The MXenes family’s main properties are discussed in this section.

#### Mechanical Properties

Mechanical features of MXenes drew a lot of attention because of the presence of strongest M–C and M–N bonds and two times higher elastic constants (c11) than MAX phases [168], and other 2D materials like MoS_2_, as per the first simulation investigation. Despite having c11 values 2 to 4 times lesser than graphene [36, 166] their bending stiffness is higher [169, 170], indicating that they could be used as composite reinforcements. Thin discs of titanium-based MXenes exhibit hydrophilic behavior with contact angles ranging from 27 to 41 degrees, whereas Ti_3_C_2_T_x_ exhibited a contact angle of 35 degrees [168]. The Young’s modulus tends to decrease as the number of layers (“*n*”) increases in both MXene carbides and nitrides [168]. Furthermore, nitride-based MXene compounds have greater values than carbides [171]. The presence of ends reduces the values of elastic constants but upsurges their critical distortions. The significantly higher values of elastic constant of MXene than graphene are a key property for flexible electronics [147]. While there are various mechanical testing methods for the characterization of bulk materials, evaluating the mechanical properties of 2D materials remains difficult. The AFM tip exerts a force at the center of a 2D MXene film in the nanoindentation technique which was used to determine the mechanical properties of 2D nanomaterials [172]. The experimental Ti_3_C_2_T_x_ monolayer Young’s modulus of 333 ± 30 GPa was obtained using this technique. Further experimental research should concentrate on developing more controllable synthesis techniques to adjust structural defects, vacancies, and different functional groups, including original molecules [170]. However, overall theoretical and practical analyses of the mechanical properties of MXene and their composites with various functionalization groups still need to be illustrated.

#### Optical Properties

Photocatalytic, optoelectronic, photovoltaic, and transparent conductive electrical devices can be made up of 2D material which absorbs in the range of visible and UV light. Ti_3_C_2_T_x_ films absorbed light in the UV–Vis ranges from 300 to 500 nm wavelength and had a transmittance of up to 91.2 percent at 5 nm thickness [173, 174]. In addition, depending on the film thicknesses, it may have a strong absorption band at roughly 700–800 nm, which causes pale greenish film color [172] and is important for photothermal diseases (PTT) treatment [175–177]. It is worth noting that the transmittance values could be improved by adjusting the thickness [178] and ion intercalation [174]. The existence of functional groups alters the optical characteristics of these 2D compounds, according to first-principles calculations [179]. In reality, unlike oxygen terminations, fluorinated and hydroxyl terminations have identical properties. When compared to pure MXene, –F, –O, and –OH terminations in MXene lower the absorption and reflectivity in the visible range, while all terminations collectively increase reflectivity in the UV range [179]. The reduction of the size of a lateral flake of MXene has recently been shown to result in decreased absorbance values [180]. A remarkable light-to-heat conversion efficiency (100%) was revealed, which could be advantageous in biomedical applications [181]. To measure the light-to-heat conversion efficiency of Ti_3_C_2_ MXene, a droplet-based light absorption and heat measurement system can be used. In this system an aqueous solution droplet (volume 9.0 μL) containing MXene is hung at the tip of a PTFE pipet (one-end-sealed), followed by a single-wavelength laser beam irradiation (473 or 785 nm), with specific power density (82 mW) and spot size (0.85 mm in diameter), right in the center of the droplet. The droplet temperature recorded by a precalibrated IR camera in real time. The total temperature profile of the droplet in response to photothermal heating and then natural cooling provides light-to-heat conversion efficiency [182, 183]. Moreover, the internal light-to-heat conversion efficiency of MXene, more specifically Ti_3_C_2_, was measured to be 100%, demonstrating a flawless energy conversion [181]. Nevertheless, to further enhance MXenes applications, several optical-associated qualities such as plasmonic, luminescence efficiency, and nonlinear optical properties must be unraveled [178–181, 184].

#### Thermal Properties

**Fig. 8:**
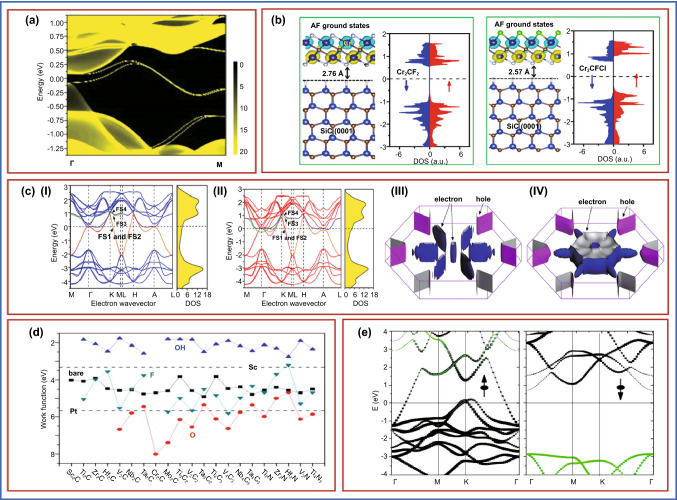
**a** Local density of states for Mo_2_HfC_2_O_2_ on the zigzag edge. The edge states connecting the bulk valence and conduction bands form a single Dirac cone at the M point. Copyright from Ref. [188]. **b** The density of states of Cr_2_CF_2_ (left panel) and Cr_2_CFCl (right panel) supported on the SiC(0001) substrate. Copyright from Ref. [192]. **c** Band structure and Fermi surface. Band structure and **(III, IV)** corresponding FS of **(I, III)** Bernal and **(II, IV)** SH Ti_3_C_2_(OH)_2_. FS1 (magenta) and FS2 (orange) partially degenerate. FS3 (green) and FS4 (violet) are partially degenerated. Copyright from Ref. [200]. **d** Work functions of MXenes with various terminations. For comparison, the work functions of Sc and Pt are also shown by dashed lines. Copyright from Ref. [201]. **e** Band structure for Cr_2_C MXene. The weights of the Cr *d* are represented in black and C *p* orbitals are represented in green. Copyright from Ref. [202]

The studies on thermal conductivities in terms of thermal expansion coefficients of MXenes are still sparse, despite their importance for electrical and energy-related heat dissipation devices [155]. Simulation studies indicated low thermal expansion coefficients [37, 184, 185] and superior heat conductivities of MXene-based materials than phosphorene and MoS_2_ monolayer [184, 186, 187]. It was observed that the thermal conductivities of oxygen–terminated compounds rise with the metal “M” atomic number [119, 184]. The edge green's function of the semi-infinite Mo_2_MC_2_O_2_ lattice is generated using the MLWFs, the imaginary component of which yields the local density of states (LDOS), from which the energy dispersion of the edge states is determined. The LDOS on the zigzag edge of Mo_2_HfC_2_O_2_ is shown in Fig. 8a, where a pair of topological edge states join the bulk conduction and valence bands to produce a single Dirac cone at the M point. Mo_2_TiC_2_O_2_ and Mo_2_ZrC_2_O_2_ produce similar results [188]. Only Ti_3_C_2_T_x_ thermal conductivity was measured in the laboratory; thus, conductivities of other MXene-based compounds should be investigated. Furthermore, the studies on the relationship between particle size and thermal conductivity that underlines the need for morphological control and optimization in MXenes synthesis need to be explored further.

#### Magnetic Properties

The MXenes can be magnetized; unlike MAX phases, several investigations carried out to evaluate their magnetic characteristics are projected to have magnetic moments. F functional groups make Ti_3_CNT_x_ and Ti_4_C_3_T_x_ non-magnetic [189], whereas OH and F groups make Cr_2_CT_x_ and Cr_2_NT_x_ ferromagnetic at ambient temperature [190], and Mn_2_NT_x_ is ferromagnetic regardless of surface terminations [191]. For Cr_2_CF_2_ and Cr_2_CFCl, the distance between MXene and substrate is 2.76 and 2.57 Å, respectively, showing only a weak van der Waals bonding. As seen in Fig. 8b, Cr_2_CF_2_ and Cr_2_CFCl on SiC(0001) retain the compensated antiferromagnetic coupling. The DOS of Cr_2_CF_2_ with a symmetrical distribution implies that there is no spin polarization, whereas Cr_2_CFCl retains the BMSAF characteristics. These findings reveal that imperfectly functionalized Janus Cr_2_CXX’ MXenes retain BMSAF properties even when they interact with the substrate, which is significant for nanoelectronic device applications [192, 193]. The reported magnetic moments are, however, simply computational predictions that have yet to be confirmed empirically. It can be due to a lack of information on surface chemistry [194] and limited synthesis of MXene compounds.

#### Electrical Properties

Several functional groups, their stoichiometry, and ability of solution formation all can be used to adjust the properties of MXenes. MXenes-pressed discs had greater electric conductivities than reduced graphene oxide and carbon nanotubes [167, 181] and were similar to multi-layered graphene. Furthermore, the number of layers in MXene and the presence of functional groups were found to improve resistivity values [195, 196]. As a result, the simulated conductivities are typically higher than those measured experimentally [197]. The defect concentration, delamination yield, d-spacing between MXenes flakes, surface functional groups, and their lateral sizes produced by each etching procedure play a crucial role in determining the electrical conductivity of MXene. The measured electrical conductivities of Ti_3_C_2_T_x_ ranged from 850 to 9880 S cm^−1^ [171, 176, 195, 196]. In general, MXenes with lower HF concentrations and etching periods have fewer flaws and bigger lateral diameters, resulting in enhanced electronic conductivity [198]. The higher flake sizes resulted in enhanced conductivities than small-sized MXenes [50]. Furthermore, relative humidity sensing material [199] may impact their conductivities. Thermal and alkaline treatments for surface modification of material are a good way to improve electrical characteristics. The change and/or alteration of functional groups (particularly F) and intercalated molecules are responsible for the rise by two orders of magnitude [178]. In momentum space, band structure describes the relationship between electronic energy and electron wavevector. The electronic band structure is the most convenient way to describe the microscopic action of electrons in a material. The band structures and density of states (DOS) in specific orientations of the Brillouin zone are depicted in Fig. 8c. The dispersion of the bands perpendicular to the basal planes (ML) is almost low, indicating that the electronic structures are essentially 2D. The conductivity is anisotropic due to the anisotropy of the band structures around and below the Fermi energy (EF). Because the electronic transport properties are determined by electrons near EF, Ti’s 3*d* electrons play a major role in electronic conduction. The form of the entire Fermi surface, which determines transport parameters such as conductivity, is visible in Fig. 8c. Four double-degenerate half-filled bands spanning EF in the band structure correspond to this. In reciprocal space, both FSs show a hexagonal electron pocket around the c* axis, surrounded by six cylindrical hole pockets. The hole-like pockets in the FSs of the two layouts are very similar: Around H and K are cylindrical hole pockets [200]. Work functions of MXenes with various terminations are shown in Fig. 8d (for comparison, work functions of Sc and Pt are depicted by dashed lines) [201]. Band structure for Cr_2_C MXene is shown in Fig. 8e. Herein, the weights of the Cr *d* are represented in black, and C *p* orbitals are represented in green [202]. The electrical characteristics of MXenes are connected to the composition of their ingredients and the number of surface termination as per density functional theory (DFT) [203]. Surface terminations of carbides are crucial being semiconductors in the MXenes family; however, some carbonitrides with additional electrons can increase band structure modification and transition to a metallic state. Ti_3_C_2_(OH)_2_ and Ti_3_C_2_F_2_ were tentatively anticipated to have modest bandgaps between 0.05 and 0.1 eV, respectively, until Yury Gogotsi and his team found MXenes [204]. Besides, mixed terminations such as –F, –OH, and –O populate the MXene’s surface at random, causing electronic state localization and altering their electrical and other characteristics. As a result, from a theoretical standpoint, modifying the element composition along with surface termination of MXenes can be used to accomplish targeted control of electrical properties. The MXene films are multilayer stacked macroscopic nanosheets for which electrical characteristics are determined by the intercalation between layers. For example, cations from reagents [205] (tetramethylammonium ion (TMA^+^), lithium-ion (Li^+^) and ammonium ion (NH_4_^+^)) and organic molecules [206] (isopropylamine and dimethyl sulfoxide (DMSO)) when intercalated into the MXenes layer, led to the modification of their electrical characteristics. As a result, post-processing modifications can successfully alter the surface termination, elemental composition, and intercalation of MXene films, allowing for focused control of their electrical properties.

## MXene-Enabled Advanced Electrochemical Aptasensors for Cancer Diagnostics

**Fig. 9:**
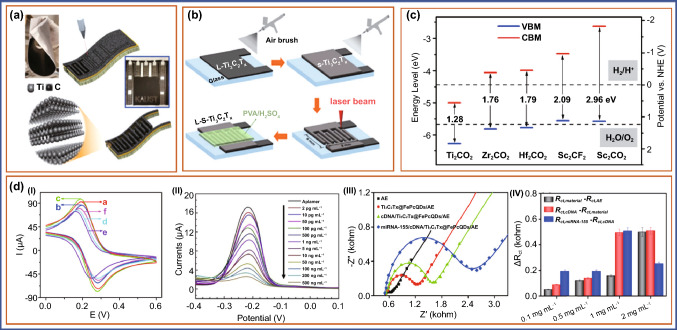
**a** Representation of the laser patterning of paper coated with MXene to form an interdigitated electrode for MSCs devices. Copyright from Ref. [213]. **b** Schematic illustrating the fabrication process of the all-Ti_3_C_2_Tx MXene MSC. Copyright from Ref. [215]. **c** The electronic band-gap for various MXenes sheets. Copyright from Ref. [214]. **d (I)** CV curves of different modified electrodes. Copyright from Ref. [166], **(II)** DPV responses to different concentrations (2 to 500 pg mL^−1^) of CEA antigens. Copyright from Ref. [216], **(III)** EIS Nyquist plots of the miRNA-155 detection in 5.0 mM [Fe(CN)_6_]^3−/4−^ containing 0.01 M PBS. Copyright from Ref. [167]. **(IV)** The corresponding variations of the *R*_ct_ values with different concentrations (0.1, 0.5, 1, and 2 mgmL^−1^) for detecting miRNA-155 detection procedures (*n* = 3). Copyright from Ref. [167]

MXene, a 2D nanomaterial with plausible electroconductive properties, has been identified as a viable molecule for the fabrication of electrochemical biosensors due to its simple manufacturing process. Aptamers, on the other hand, have proven to be a boon for manufacturing low-cost sensing devices due to their great selectivity and specificity, as well as their mass production ability. The advantageous electroconductive properties of MXene enabled with selective and specific aptamers against cancer-specific biomarkers can be potentially employed for early and efficient diagnostics of cancer which is the need of an hour. In this section, we have discussed the studies on MXene-enabled electrochemical aptasensors for the detection of cancer-specific biomarkers. For the synthesis of various types of MXene composite, Naguib et al. added the Ti_2_C, Ta_4_C_3_, TiNbC, (V_0.5_, Cr_0.5_)_3_C_2_, and Ti_3_CN_x_, where *x* < 1 which expands the MXenes family [207]. In further work, Naguib et al. proved that MXenes can also be used as electrodes when intercalated with Li ions in lithium-ion batteries [208]. Aptamer-enabled MXene-based electrochemical biosensors have been used in recent studies to detect a variety of cancer biomarkers, as stated in Table 2. In a recent study, the breast cancer marker Mucin 1 was detected using a competitive electrochemical aptasensor fabricated on a cDNA-ferrocene/MXene probe (MUC1). Herein, MXene (Ti_3_C_2_) nanosheets with high specific surface area and excellent electrical conductivity were chosen as aptamer-probe carriers. To make a cDNA-Fc/MXene probe, ferrocene-labeled complementary DNA (cDNA-Fc) was coupled to the surface of MXene followed by attachment of MUC1 aptamer on the electrode via Au-S bonds. A cDNA-Fc/MXene/Apt/Au/GCE aptasensor was made and utilized to detect MUC1 through a competitive process that occurs between the cDNA-ferrocene/MXene probe and MUC1. The reduction in an electrical signal happens due to the detachment of the cDNA-Fc/MXene probe from the sensing electrode. This aptasensor has a broad linear range of 1.0–10 µM and a LOD of 0.33 pM, making it suitable for clinical diagnostics [209]. An electrochemiluminescent (ECL) aptasensor for detection of exosomes in breast cancer cells is described by Qiao et al. ECL emitters and reactants used in this study were H_2_O_2_ and mercaptopropionic acid (MPA)-modified Eu^3+^-doped CdS nanocrystals (MPA-CdS: Eu NCs), respectively. The CD63 aptamer recognizes and captures exosomes, which subsequently create a G-quadruplex/hemin DNAzyme that competently causes the breakdown of H_2_O_2_, and thereby reduced the ECL signal in MPA-CdS: Eu NCs. The exosomes from breast cancer cells (MCF-7cells) can be found in concentrations ranging from 3.4 × 10^5^ to 1.7 × 10^8^ particles mL^−1^. The LOD and signal-to-noise ratio was determined to be 7.41 × 10^4^ particles mL^−1^. Exosomes in the serum have been effectively detected using this aptasensors [210]. In a similar study, CD63 aptamer-modified poly(amidoamine) (PAMAM)-Au NP electrode interface which has a high binding affinity for CD63 protein on exosomes generated from OVCAR cells has been fabricated for detection of exosomes. Furthermore, the CD63-modified Ti_3_C_2_ MXene was employed as a nanocarrier for several aptamers and was adsorbed to exosomes. The Ti_3_C_2_ MXene is generated in situ and loads it efficiently, as well as magnifies the electrochemical signal at a low potential, minimizing interference from the electrochemically active species. This aptasensor shows a linear range of 5 × 10^2^ particles µL^−1^ to 5 × 10^5^ particles µL^−1^, and the LOD was 229 particles µL^−1^. This electrochemical aptasensor can detect exosomes from a variety of cancer cells, including OVCAR, HeLa, and BT474, and in serum samples with high specificity suggesting its clinical diagnostic potential early cancer detection [211]. The nanohybrid of Ti_3_C_2_T_x_ MXene and phosphomolybdic acid (PMo12) embedded with polypyrrole (denoted as PPy@Ti_3_C_2_T_x_/PMo12) was synthesized by Zhou et al. Further it was attached to osteopontin (OPN) aptamer to build an impedimetric aptasensor for the detection of osteopontin. The fabricated sensor PPy@Ti_3_C_2_T_x_/PMo12 hybrid is endowed with outstanding stability, great biocompatibility, and a significant binding affinity for OPN aptamer. Thus, compared to previously developed bicomponent aptasensors, the PPy@Ti_3_C_2_T_x_/PMo12 hybrid demonstrated improved electrochemical sensing. The aptasensor based on PPy@Ti_3_C_2_T_x_/PMo12 had a detection limit of 0.98 fg mL^−1^, as well as high selectivity and stability, better repeatability, reasonable regenerability, and can be used to detect OPN in human serum samples [212]. The direct laser patterning of various coplanar of MXene on the paper device and multiple devices with series and parallel connections can be fabricated using hydrofluoric acid (HF)-etched and clay-like Ti_3_C_2_ MXene slurries in just 17 s using additive manufacturing technique (Fig. 9a) [213]. The fabrication steps of on-chip MXene solid-state micro-supercapacitors (MSCs) by employing a spray-coating method for deposition of highly conductive Ti_3_C_2_Tx (L-Ti_3_C_2_T_x_) flakes on a glass substrate were demonstrated by Peng et al. (Fig. 9b). MXene-based composite meets the requirement of the bandgap value which should be between 1.55 and 3.0 eV (Fig. 9c) [214]. They used four steps which include spray coating of small-size Ti_3_C_2_T_x_ flakes (s-Ti_3_C_2_T_x_) on top as an electroactive layer, interdigital pattern carved by direct laser cutting of a specific center area (8 × 6 mm^2^) on the stacked MXene film, and a PVA/H_2_SO_4_ gel electrolyte was carefully dropped onto the interdigital pattern area, respectively [215]. Employing this process would enable the sensor fabrication with ease and in a cost-effective manner. Fang et al. used black phosphorous quantum dots (BPQDs) and MXenes as a signal amplifier for fabrication of ECL and photothermal dual-mode aptasensor for detection of the cancer-associated exosome. Herein, BPQDs catalyze the oxidation of Ru(dcbpy)_3_^2+^ and be utilized as a co-reactant. The self-enhanced Ru(dcbpy)_3_^2+^@BPQDs ECL system generates a strong ECL signal by shortening electron transfer distance and minimizing energy loss. MXenes provide large specific surface area and excellent conductivity and act as a supporter to enhance the number of Ru(dcbpy)_3_^2+^ and BPQDs immobilized, which improved the ECL signal. They studied the cyclic voltammetry (CV) behaviors of ECL biosensor over electrodes in 5 mM [Fe(CN)_6_]^3−/4−^ including 0.1 M KCl. As shown in Fig. 9d(I), bare GCE displayed a pair of well-defined redox peak currents (curve a); however, when SiO_2_ nano urchins (NUs) were placed over the electrode, the redox peak current diminished (curve b), which was linked to SiO_2_ NUs with weak conductivity and hence blocking electron transport. The redox peak currents dramatically increased when ILs were coated onto the electrode due to the promotion impact of ILs for electronic transmission (curve c). The redox peak currents reduced dramatically (curve d) after Apt was incubated on the electrode, which was attributed to the aptamer with negatively charged phosphate backbone inhibiting the diffusion of the redox probe [Fe(CN)_6_]^3−/4−^ to the electrode surface. In exosomes modified electrodes, the redox peak currents are reduced much further (curve e). In contrast to (curve f), when MXenes-BPQDs@Ru(dcbpy)_3_^2+^-PEI-AbCD63 (curve f) was deposited onto the electrode, the redox peak currents increased dramatically, indicating that MXenes and BPQDs have a synergistic promotion effect for electron transfer (Fig. 9d(I)). Additionally, both BPQDs and MXenes have a good photothermal effect, which was deftly exploited as a thermal converter device in the development of a photothermal biosensor for exosome characterization. The developed dual-modality MXenes-BPQDs probe aptasensor in this study not only enhanced the signal while detection but also provided an effective and reliable approach for exosome detection in cancer patients [166]. A label-free, ultrasensitive, and multiplexed microfluidic paper-based electrochemical aptasensor for simultaneous detection of carcinoembryonic antigen (CEA) and neuron-specific enolase (NSE) was developed by Wang et al. which shows LOD 2 pg mL^−1^ for CEA and 10 pg mL^−1^ for NSE. The degree of decreased peak currents in DPV responses, which was attributable to the formation of aptamer antigen complex on the electrode surface, was used to detect the two analytes. The currents steadily reduced when CEA (3 A) and NSE increased, as shown in Fig. 9d(II). In the ranges of 0.01 ~ 500 ng mL^−1^ for CEA (*R*^2^ = 0.989) and 0.05 ~ 500 ng mL^−1^ for NSE (*R*^2^ = 0.944), the calibration plots revealed a satisfactory linear detection relationship between peak currents and analyte concentrations [216]. Recently, iron phthalocyanine quantum dots (FePcQDs) decorated MXene nanosheets (denoted as Ti_3_C_2_T_x_@FePcQDs) and employed as promising nanocarrier of complementary DNA (cDNA) toward miRNA-155. This construct was used as a novel ultrasensitive impedimetric autosensing system for the detection of microRNA-155 (miRNA-155). The Ti_3_C_2_T_x_@FePcQDs-based aptasensor demonstrated ultrahigh sensitivity with LOD (limit of detection) of 4.3 aM (S/N = 3) within the miRNA-155 concentration range of 0.01 fM to 10 pM. for miRNA-155 detection; the suggested impedimetric autosensing system outperformed other published miRNA-155 aptasensors in terms of ease of fabrication, lack of labels, fast reaction time, and better sensing performance for detecting miRNA-155. This technique for determining cancer-associated miRNAs holds a lot of potential for early cancer biomarker detection [166]. Electrochemical impedance spectroscopy (EIS) Nyquist plots of miRNA-155 detection techniques employing an electrochemical aptasensor based on a Ti_3_C_2_T_x_@FePcQDs nanohybrid in 5.0 mM [Fe(CN)_6_]^3−/4−^ in 0.01 M PBS are shown in Fig. 9d(III) [167]. The experimental parameters specifically the use of Ti_3_C_2_T_x_@ FePcQD nanohybrid, cDNA concentration, and miRNA-155 binding time onto the Ti_3_C_2_T_x_@FePcQDs-based aptasensor were tuned to produce excellent sensing performance for miRNA-155 detection. EIS was used for the entire fabrication and detecting process. The R_ct_ values generated by each step for miRNA-155 detection ( fM) utilizing aptasensors based on the Ti_3_C_2_T_x_@FePcQD nanohybrid with varied usages (0.1, 0.5, 1.0, and 2.0 mg mL^−1^) under the same conditions are shown in Fig. 9d(IV). The results showed that as the concentration of the Ti_3_C_2_T_x_@FePcQDs dispersion grew from 0.1 to 1 mg mL^−1^, the *R*_ct_ values originating from the detection of miRNA-155 increased. When the Ti_3_C_2_T_x_@FePcQDs dispersion concentration was more than 1 mg mL^−1^, the *R*_ct_ value for detecting miRNA-155 dropped dramatically [167].

**Table 2:** MXene-based electrochemical aptasensors for cancer diagnosis (sensitivity and detection limit)

S. no.	Material	Biomarker	Sample	Technique	Linear range	LOD	References
1	MXenes-BPQDs@Ru(dcbpy)_3_^2+^-PEIAb_CD63_	Exosomes, CD63		CV	1.1 × 10^2^ to 1.1 × 10^7^ particles µL^−1^	37.0 particles µL^−1^	[166]
2	0D/2D Ti_3_C_2_T_x_@FePcQD nanohybrid	miRNA-155	Serum	EIS	0.01 fM to 10 pM	4.3 aM	[167]
3	cDNA-Fc/MXene/Apt/Au/GCE aptasensor	Mucin 1	Serum	SWV	1.0 to 10 µM	0.33 pM	[209]
4	MPA-CdS:Eu NC	Exosomes, CD63	Serum	EIS, ECL	3.4 × 10^5^ to 1.7 × 10^8^	7.41 × 10^4^ particles mL^−1^	[210]
5	Ti_3_C_2_ MXene (MXene)	Exosomes, CD63	Cancer cells and Serum	CV, EIS	5 × 10^2^ to 5 × 10^5^ particles µL^−1^	229 particles µL^−1^	[211]
6	PPy@Ti_3_C_2_Tx/PMo12	Osteopontin	Serum	EIS	–	0.98 fg mL^−1^	[212]
7	AuNPs/Ti_3_C_2_ MXene	miRNA-155		EIS, CV	1.0 fM to 10 nM	0.35 fg	[217]
8	MXenes-Apt2/exosomes/Apt1/PNIPAM-AuNPs/GCE	Exosomes, CD63	Serum	ECL	5.0 × 10^2^ to 5.0 × 10^6^ particles µL^−1^	125 particles µL^−1^	[218]
9	MXene-MoS_2_-Thi-AuNPs/GCE	miRNA-21	Serum	CV, EIS, SWV	–	26 fM	[219]
10	CoFe_2_O_4_@Ag-HB5 cytosensor	HER2 positive cells	Blood sample	CV, EIS	10^2^ to 10^6^ cells mL^−1^	47 cells mL^−1^	[220]
11	eCoCu-ZIF@CD-based cytosensor	PTK7B16-F10 cells		EIS, CV	1.0 × 10^2^ to 1.0 × 10^5^ cells mL^−1^	33 cells mL^−1^	[221]

An electrochemical aptasensor employing an AuNPs/Ti_3_C_2_ nanocomposite for sensitive detection of miRNA-155 using Exonuclease III (Exo III)-assisted cascade has been developed by Yang et al. AuNPs utilize AuS chemical bonds to immobilize capture DNA (C-DNA) on which methylene blue (MB) was tagged at the 3′ end of the C-DNA. MiRNA-155 double-stranded structure by complementary base pairing with C-DNA upon which Exo III catalyzes digestion of the double-stranded C-DNA. This led to the electrochemical signal to “switch off.” The developed sensor exhibits a linear range of 1.0 fM to 10 nM and LOD of 0.35 fg. (S/N = 3). In addition, the developed sensor has good stability, repeatability, and specificity [217]. Owing to the MXenes properties such as large surface area, excellent conductivity, and catalytic properties, Zhang et al. developed sensitive electrogenerated chemiluminescence (ECL) biosensor for the detection of the exosome. They used aptamer-modified 2D Ti_3_C_2_ MXenes nanosheets as the ECL nanoprobe. An aptamer against EpCAM protein was modified on the surface of the electrode for the capture of the exosome. This ECL nanoprobe showed much-improved luminol ECL signals. The detection limit of this aptasensor was 125 particles L^−1^, which is more than 100 times lower than the detection limit of a standard ELISA approach. The exosomes in the serum were successfully detected using this ECL biosensor and hence can be implemented in clinical diagnostics [218]. Liu et al. combined a 2D bimetallic CoCu-zeolite imidazole framework (CoCu-ZIF) with 0D Ti_3_C_2_T_x_ MXene-derived carbon dots (CDs) and termed it CoCuZIF@CDs. It showed a unique heterogeneous architecture and possesses a sensitive layer for attachment of B16-F10 cell-targeted aptamer strands, hence detecting B16-F10 cells in the biological sample. The characterization showed that CDs were uniformly embedded into CoCu-ZIF NSs with appropriate stacking interaction. This led to enhanced fluorescence performance of 0D/2D CoCu-ZIF@CD nanohybrids. The developed electrochemical aptasensor can be used for cell imaging and detection of living B16-F10 cells. The CoCu-ZIF@CD-based cytosensor exhibits LOD of 33 cells mL^−1^ and linear range of detection from 1.0 × 10^2^ to 1.0 × 10^5^ cells mL^−1^. In comparison, the CoCu-ZIF@CD-based cytosensor displayed better performance when compared to CoCu-ZIF and CD-based cytosensors. The cell imaging properties, outstanding selectivity, high stability, and good repeatability of developed CoCu-ZIF@CD-based aptasensor can be exploited for early diagnosis of other analytes too by anchoring other probe molecules, hence expanding its applications in biosensing and biomedical domains [66]. A label-free determination of microRNA-21 (miR-21) was successfully demonstrated using an MXene-MoS_2_ heterostructure-based electrochemical biosensor conjugated with catalytic hairpin assembly (CHA) amplification. The large specific area and better electroconductivity offered by this unique micro-nanoheterostructure enhance the sensing performance. This MXene-MoS_2_ heterostructure triggers more target recycling reactions when compared to traditional CHA amplification approaches. Besides, the anchored thionine and gold nanoparticles (AuNPs) over the surface of MXene-MoS_2_ heterostructure further empowered the sensor performance in terms of probe capture fixation and label-free detection of miR-21. In the detection process, several electronegative double-stranded DNA was generated which hindered the electron transfer resulting in a decrease of a signal. This sensor showed a broad linear range from 100 fM to 100 nM and LOD of about 26 fM. However, this sensor is stable, reproducible, and selective for miR-21 detection and also provides satisfactory and reproducible results. However, the sensing performance of this aptasensor for the detection of miR-21 is found to be either comparable or lower than previous methods even though it showed a promising performance under clinical conditions [219]. In recent research work, Vajhadin et al. developed an aptasensor for electrochemical detection of tumor cells by using HER-2 biomarker. For the development of an aptasensor, the MXene nanosheets of around 2 nm thickness and 1.5 μm lateral size were fabricated over gold electrodes. An HB5 aptamer that shows high selectivity for HER-2-positive cancer cells was then immobilized on the MXene layers. To minimize biofouling of electrode with blood matrix, CoFe_2_O_4_@Ag magnetic nanohybrids bonded to the HB5 were used for magnetic separation of HER-2-positive cancer cells. The magnetically captured cells formed sandwich-like structures with MXene-functionalized electrodes which effectively blocks electron transfer and allows quantitative cell detection when current signal changes. This label-free MXene-based aptasensor exhibited a wide linear range of 10^2^–10^6^ cells mL^−1^ and a LOD of 47 cells mL^−1^. Additionally, it provides decent sensitivity and selectivity against HER2-positive cells detection in blood samples. Therefore, this CoFe_2_O_4_@Ag magnetic nanohybrids and MXenes-based aptacytosensor hold promise to screen cancer progression cost-effectively [220]. The MXene-based 2D material when used in combination with aptamer as biorecognition element for the fabrication of electrochemical aptasensor has enhanced the sensitivity of detection of cancer-associated biomarkers many folds with a linear range of detection as revealed by several studies discussed in this section. However, this field is still in its nascent stage and needs to be explored further in terms of clinical validation of aptasensors in hospitals. Validation of these aptasensors in large cohorts under clinical settings would have the potential to revolutionize the field of cancer diagnostics and needs further attention. The challenges and future perspectives in this regard are discussed in Sect. 6.

## Current Challenges and Prospects

Since the discovery of MXene in 2011, MXenes are widely used in different fields including diagnostic and therapeutic. Although MXene is used widely, some lacunae need to be fulfilled to harvest its full potential. Although their 70 + MXene are known, the number is growing rapidly. There is the possibility to explore many more compounds which are waiting to be included in the MXene family. The synthesis of new MAX phases and other layered carbide and nitride precursors is a hot research direction. The prediction of the various electronic, magnetic, thermal, and chemical properties to get the tunable size, ordered structures, strong surface terminations, and high yielding is needed. This motive can be achieved using computational strategies which can further increase the feasibility of MXene synthesis. With advanced knowledge, the ion dynamics between the sheets of MXene can be controlled or altered to obtain the desired electronic conductivity of the MXene. This can be utilized to fabricate sensors with higher conductivity and lowest resistance to obtain low LOD. Understanding the electrical properties of the MXene can pave new paths in the research field. Very recently, the ionic gel-based highly durable electronic skins were fabricated utilizing ionic gel and MXenes embedded into the polymer matrix. The electronic skin showed excellent mechanical properties, super adhesion, high sensitivity to strain and pressure and could tolerate harsh environment. Owing to the various excellent properties, the fabricated skin can be applied for the multifunctional sensing purposes [193]. Similarly, for serving the healthcare monitoring purpose, the strain sensor was fabricated. The sensor was based on the carbon nanotubes and MXenes into polydimethylsiloxane matrix. The sensor was found to be highly stable, durable, and moreover washable, and could be used for the real-time monitoring of the electrocardiogram (ECG) and joints movements [39]. These types of wearable sensor need to be explored more and utilized for the healthcare monitoring purposes. The conventional synthetic approach of MXene from the MAX phase includes the use of fluoride-containing compounds. The fluoride-containing compounds pose safety hazards and also limit the yield of MXene as it can alter the synthesis of MXene from the Al-containing MAX phase. The fluoride-free approach using hydrochloric acid was also used for the electrochemical etching of the MXene from the MAX phase, but the over-etching and scaling up remain the challenge. The search for new etching methods has become a frequent topic of research among scientists working in the area of synthesis, so major developments can be expected soon. The main challenge is to develop a sensitive, easy-to-use, cost-effective Point-of-Care-Test (POCT) to eradicate the false-negative and false-positive results.

Certain points limit the use of MXene practically. Firstly, strong acids are used for the exfoliation as the MAX phase layers have strong interactions. The use of these strong acids restricts the use of MXenes in various fields, especially in green chemistry. The other harsh chemicals and use of sophisticated instruments for the synthesis are also a limiting point. This point raises the demand for the new etching methods and experimental conditions which use alternative chemicals. Despite the excellent features, several issues limit the practical applications of MXenes. The majority of the problems arise due to the synthesis process that requires the use of toxic chemicals and harsh experimental conditions. Hence, new experimental routes and conditions should be explored to synthesize novel MXenes with unique properties. In this regard, recently MXene quantum dots were synthesized using watermelon peel extract. The synthesized material was utilized for the biocompatible sensing purposes and also integrated with the smart phone. The more similar work in this field is expected in future for the sensing of various analytes and fabrication of wearable sensor [51]. The use of M in the MAX phase is only limited to some elements Ti, V, Nb, Mo, Ta, Hf. However, many more elements can be used as M in MAX which needs to explore. Instead of metal carbides, metal nitrides can also be used. Till now, there are no more studies available on the different morphology of the MXenes; only the sheet is broadly studied even though other morphology like tube spheres also exists. The complex structure of MXenes during synthesis is still a challenge for researchers [221]. For understanding MXene better, due to its multivariate structure, further studies are needed. Computational approaches can pave the way for designing new MXenes structures. MXenes family can be further expanded by designing and discovering new heterostructures. The search for new etching methods has become a frequent topic of research among scientists working in the area of synthesis, so major developments can be expected soon. The various work has been already done on the MXenes, but still, there is a long way to go. Researchers are working on the greener synthesis of MXenes for replacing the harsh chemical. By exploring the various properties of MXenes like superconductivity, thermal transport, biocompatibility, etc., it can be exploited to the various multidisciplinary fields. The MXenes displayed tunable magnetic and electric properties and used as an excellent modifier in electrochemical studies and hence showed adaptable applications in various fields such as energy storage, nanomedicine, diagnosis [198]. MXene-enabled aptasensors have shown considerable promise for the early detection of cancer biomarkers. Aptamers have gained much attention in the last few decades and are explored for diagnostic and even therapeutic purposes. The ease of synthesis, low cost, and stability, make aptamer preferable candidate over other BREs. Aptamers also have many pitfalls including difficult multianalyte detection, cross-reactivity, poor precision, etc. The limiting factor in the designing of aptamers is the library design. It usually depends on the oligonucleotide’s quality and length, its structural stability, specific binding sequences. The quality of the oligonucleotides depends on the nucleotide’s ratio and the level of complexity of an aptamer library. More and more research in the field to make aptamer preferable, cost-effective has improved its demand. More research is in demand for better integration and detection of cancer biomarkers. Advanced bioinformatics techniques and genetic algorithm-based methods are required for studying multiple cancer cells and studying the binding affinities of biomarkers with specific aptamers. Major challenges which need to be addressed include the methods to screen aptamer rapidly with high specificity, maintaining stability in the biological environment, reducing the toxicity of nanomaterials used in conjugation with aptamers. The MXene and aptamer-based devices can be commercialized as POCT devices. Current approaches for the diagnosis of various diseases have been using a lateral flow-based system either in the form of electrical or optical-based devices. The major challenge is device miniaturizations and multiplexing. POCT devices should be affordable, sensitive, specific, user-friendly, robust and can always be performed outside a laboratory or hospital by a non-technical person or by a layman as per ASSURED criteria. Other than cost-cutting, the POC test devices should give a signal in a lesser sample volume (microliter). The device that can differentiate between different biomolecules and can be used for multiplex analyte sensing is the need of the hour. MXene enabled electrochemical aptasensors to have the potential to fulfill the criteria for effective POCT devices for early disease diagnostic.

**Fig. 10:**
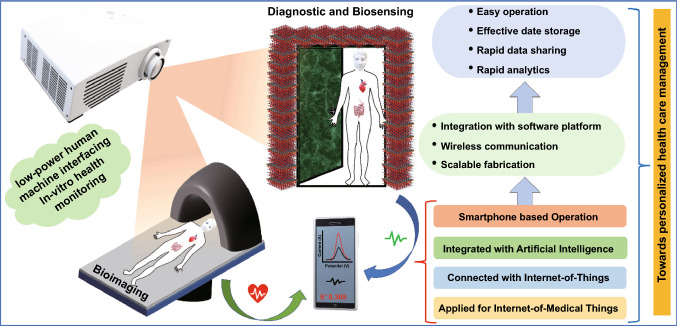
Futuristic application of MXene toward personalized healthcare management

There is an enormous interest in AI-integrated POCT devices to improve treatment efficacy and health care. A convolutional neural network (CNN) is one of the important AI-based tools that can be integrated with devices for the prediction of disease in resource-limited settings. These devices can also be interlinked with the top-class portable healthcare interfaces such as smartphones, smartwatches, and other wearable devices. These devices can also be used to monitor the healthy as well as the patient’s conditions. By integrating these devices with the IoT and IoMT devices, the past and present records of the individual’s health condition can be assessed by medical practitioners for better disease prognosis and decision making [222, 223]. These devices can be used to forecast clinical conditions digitally. This will be a rapid, cost-effective, intelligent approach to deal with the disease condition even in resource-limited settings. The detailed insight of MXene-enabled healthcare diagnostics coupled with IoT and IoMT and smartphones and their futuristic application are illustrated in Fig. 10. With the interdisciplinary amalgamation of various fields such as material chemistry, biomedical engineering, computational chemistry, the MXene enabled electrochemical aptasensor to have the potential to tackle the challenges associated with the fabrication and development of POCT devices for early cancer diagnostics.

## Concluding Remarks

Despite the various advancements in the field of oncology, cancer still poses an enormous risk and challenges to tackle. There are several challenging factors which are needed to be addressed and resolved. This review emphasizes of MXene, MXene-based materials, and their conjugation with aptamers for the fabrication of electrochemical aptasensor for cancer diagnostics with a focus on the current trends, important hurdles, and their future insights. The role of nanotechnology in dealing with deadly diseases such as cancer is important. Many portable biomedical devices for imagining, sensing, and other purposes were successfully developed using nanomaterials. MXene is a new class of nanomaterial emerging at a high rate. MXene is significantly used for diagnostic and other biomedical purposes. Many advancements have been made globally by many investigators to explore the properties of MXene. On the other hand, aptasensors as POCT have achieved significant attention in the past few decades. The aptamers have remarkable advantages over the conventional biorecognition elements. The aptasensors are economical, stable, sensitive, selective, and show negligible batch-to-batch variation. This review focused on MXene-enabled electrochemical aptasensors for the detection of cancer biomarkers. However, the validation of these aptasensors in a larger patient cohort using a real sample is still needed. Further, challenges associated with device miniaturization, multiplexing, and its integration with IoT-enabled smartphones need to be resolved by using an amalgamation of interdisciplinary fields to optimize the POCT sensing systems. The development of MXene-enabled electrochemical aptasensor for the real-time, cost-effective, and early diagnosis of cancer still has a long way to go. Nevertheless, there are many hurdles in the commercial utilization of the MXene-based aptasensors; however, further research in this field will prepare the base for the commercialization of the MXene-based aptasensors for cancer diagnostics.
